# Oxygen therapy in patients with ST elevation myocardial infarction based on the culprit vessel: results from the randomized controlled SOCCER trial

**DOI:** 10.1186/s12873-020-00309-y

**Published:** 2020-02-18

**Authors:** Arash Mokhtari, Mahin Akbarzadeh, David Sparv, Pallonji Bhiladvala, Håkan Arheden, David Erlinge, Ardavan Khoshnood

**Affiliations:** 1 Department of Clinical Sciences Lund, Cardiology, Lund University, Skåne University Hospital, Lund, Sweden; 2 Department of Clinical Sciences Lund, Emergency and Internal Medicine, Lund University, Skåne University Hospital, Akutmottagningen, EA10, SUS Lund, 221 85 Lund, Sweden; 3 Department of Cardiology, Skåne University Hospital, Malmö, Sweden; 4 Department of Clinical Sciences Lund, Clinical Physiology, Lund University, Skåne University Hospital, Lund, Sweden

**Keywords:** ST elevation myocardial infarction, Oxygen, Myocardial infarction, Left anterior descending artery, Cardiac magnetic resonance imaging, Myocardium at risk, Infarct size, Myocardial salvage index, High-sensitive troponin T, Randomized controlled trials

## Abstract

**Background:**

Oxygen (O_2_) treatment has been a cornerstone in the treatment of patients with myocardial infarction. Recent studies, however, state that supplemental O_2_ therapy may have no effect or harmful effects in these patients. The aim of this study was thus to evaluate the effect of O_2_ therapy in patients with ST Elevation Myocardial Infarction (STEMI) based on the culprit vessel; Left Anterior Descending Artery (LAD) or Non-LAD.

**Methods:**

This was a two-center, investigator-initiated, single-blind, parallel-group, randomized controlled trial at the Skåne university hospital, Sweden. A simple computer-generated randomization was used. Patients were either randomized to standard care with O_2_ therapy (10 l/min) or air until the end of the primary percutaneous coronary intervention. The patients underwent a Cardiac Magnetic Resonance Imaging (CMRI) days 2–6. The main outcome measures were Myocardium at Risk (MaR), Infarct Size (IS) and Myocardial Salvage Index (MSI) as measured by CMRI, and median high-sensitive troponin T (hs-cTnT).

**Results:**

A total of 229 patients were assessed for eligibility, and 160 of them were randomized to the oxygen or air arm. Because of primarily technical problems with the CMRI, 95 patients were included in the final analyses; 46 in the oxygen arm and 49 in the air arm. There were no significant differences between patients with LAD and Non-LAD as culprit vessel with regard to their allocation (oxygen or air) with regards to MSI, MaR, IS and hs-cTnT.

**Conclusion:**

The results indicate that the location of the culprit vessel has probably no effect on the role of supplemental oxygen therapy in STEMI patients.

**Trial registration:**

Swedish Medical Products Agency (EudraCT No. 2011–001452-11) and ClinicalTrials.gov Identifier (NCT01423929).

## Background

In a letter in 1859, Dr. Birch stated that even though oxygen therapy has an important role in the field of medicine, it should be used with caution [1]. Four decades later, Dr. Charles Steele, presented a case in which a patient with suspected angina had a relief of chest pain when treated with supplemental oxygen (O_2_) [2]. Ever since, supplemental O_2_ therapy has been an important treatment in patients with chest pain and suspected myocardial infarction (MI).

Because of contradicting results and small studies, the cardiovascular effects of supplemental O_2_ therapy in canines, healthy individuals as well as individuals with MI was for a long time unclear [3]. Recent randomized controlled trials (RCT) on patients with suspected [4–6] as well as confirmed MI [7] and ST Elevation Myocardial Infarction (STEMI) [8–14] have however shown no positive nor negative cardiovascular effects of supplemental O_2_ therapy. Consequently, both the current guideline of the European Society of Cardiology for the management of STEMI [15], as well as the recently published British Medical Journal rapid recommendation on oxygen therapy [16], recommend that supplemental O_2_ therapy should not be administrated in patients with MI and a blood oxygen saturation of ≥90%.

None of the above RCTs have however evaluated the effects of supplemental O_2_ therapy in patients with STEMI based on the culprit vessel. It has been shown that Left Anterior Descending (LAD) infarction results in both a larger myocardium at risk and a larger infarct size [17] in comparison to non-LAD infarctions. As supplemental O_2_ therapy has been suggested to increase oxygen delivery [18–22], it could thereby theoretically have a role in LAD infarcts. There are also studies that have shown potential harm with oxygen therapy with reduced coronary blood flow [23–26], why an adverse effect on infarct size could also be considered. It is therefore unknown whether O_2_ therapy might have a role in patients where the culprit vessel is the LAD artery. In this sub-study of the Supplemental Oxygen in Catheterized Coronary Emergency Reperfusion (SOCCER) trial, we therefore aimed to study the effects of supplemental O_2_ therapy in STEMI patients with regard to Myocardial Salvage Index (MSI), Myocardium at Risk (MaR), and Infarct Size (IS) based on whether the culprit vessel was the LAD or not. Our hypothesis was that O_2_ therapy would not provide a significant benefit even in LAD infarcts.

## Methods

The current research is a sub-study of the SOCCER trial. The trial was a two-center, investigator-initiated, single-blind, parallel-group, randomized controlled trial and was carried out between 23 January 2012 and 5 August 2015 at the Skåne University Hospital in Lund and Malmö in Region Skåne, southern Sweden.

The trial was approved by both the Swedish Medical Product Agency and the Regional Ethical Review Board in Lund.

The current sub-study is reported in accordance with the CONSORT statement [27]. The methods of the trial will only be briefly discussed and described, since it has been described in detail previously [10, 11, 28].

### Study design and study setting

The Skåne University Hospital in Malmö and Lund each have a 24-h emergency department with a total patient census of 150,000 annually.

After being dispatched to a patient with chest pain, the ambulance personal record an electrocardiogram (ECG) and transmit it to the nearest Cardiac Care Unit (CCU) where the physician on call interprets it. If deemed to be a STEMI, the patient is brought directly to the catheterization laboratory for Primary Percutaneous Coronary Intervention (PPCI). If the angiogram showed no lesion thus no PPCI was conducted, the patient would still be included in the final analysis.

The trial planned to include 100 patients. Inclusion criteria were normoxemic (blood O_2_ saturation ≥ 94%) first time STEMI patients accepted for PPCI with a symptom duration of less than 6 h, also being able to give an informed consent. Exclusion criteria were subsequently inability to make any decision of participating, previous myocardial infarction as well as, in regard to CMRI, significant claustrophobia or having magnetic materials inside the body. The patients were randomized in the ambulance to either standard O_2_ therapy in accordance with the ambulance guidelines in Region Skåne (10 l O_2_/min) or no supplemental O_2_ therapy to be given through an OxyMask until the end of the PPCI. On days 2–6 after the PPCI, all included patients underwent a CMRI to determine MaR, IS and MSI.

Patients were randomized 1:1 to one of the two arms using block sizes of six. The blocks contained information on patient allocation based on computer-generated random numbers.

### Patient management

Both the ambulance personnel as well as the PCI laboratory personnel registered patient management on case report forms. Other in-hospital data including the results of the PPCI were retrieved from patient records of Region Skåne (Melior; Siemens, Erlangen, Germany) and from the SWEDEHEART quality registries RIKS-HIA and SCAAR (http://www.ucr.uu.se/swedeheart/).

Except for the randomization and thus treatment with supplemental or no supplemental O_2_, all patients were treated and managed in accordance with international and national guidelines for the treatment of STEMI in both the pre-hospital and in-hospital setting [29–31].

#### Culprit vessel

The culprit vessel was defined as the coronary artery responsible for the ischemic event as identified by the interventional cardiologist performing the coronary angiography.

As the results of the angiography are not available to the physician upon first medical contact, the infarct location as determined by the initial ECG were also evaluated [32]. For this analysis the infarct location was divided into anterior/anteroseptal/anterolateral (AAA) or non-anterior/anteroseptal/anterolateral (N-AAA), with AAA assumed to be due to LAD culprit, and N-AAA to non-LAD. The initial ECG assessment by the CCU physician was retrieved. All ECGs were then also independently interpreted by one of the study authors (AK) and if not in accordance with the interpretation of the CCU physician, they were analyzed by another of the study authors (AM). Both AK and AM were blinded to the group allocation of the patients.

#### Cardiac magnetic resonance imaging

Included patients underwent CMRI in either Lund (Philips 1.5 T Achieva, Best, Netherlands or Siemens 1.5 T Aera, Erlangen, Germany) or Malmö (Siemens 1.5 T Avanto, Erlangen, Germany) in two-chamber, four-chamber and LV outflow tract views (long-axis images) as well as short-axis images covering the LV. Details of the CMRI have previously been published [11].

In short, we quantified the MaR (the ischemic area before the PCI), the IS, and the MSI calculated as (1 – IS/MaR) × 100%. Images from the CMR were assessed in the short-axis images after administration of gadoteric acid.

There are different methodologies in quantifying the infarction in images from the CMR. We used a validated semi-automatic algorithm [33] showing no bias when compared to histochemical staining 7 days after a MI [34]. The images were analyzed using the postprocessing software Segment, v.1.9 R3084 [35] by a physician blinded to all data and the patient’s study group allocation.

The primary outcome of the study was MSI. Secondary outcomes were MaR, IS and median peak high sensitivity troponin-T (hs-cTnT). We chose MSI as the primary endpoint as it has been shown to be highly related to the prognosis of a STEMI patient undergoing PPCI [36] and allows the evaluation of the effects of therapies using a smaller sample size [37].

### Statistical analysis

For descriptive data, continuous variables are described with mean and standard deviation or median with interquartile range, and categorical variables are described with proportions.

The two arms of the trial were each divided into two sub-groups with respect to the culprit vessel; LAD and non-LAD. All data were analyzed using IBM SPSS Statistics (V22; Armonk, New York, USA). For differences in CMRI results between the two arms of the study, the two-sided Mann-Whitney test were used. To model the effects, linear regression with an interaction term was used. A *p*-value of less than 0.05 was considered to be statistically significant.

A power calculation for the main outcome of the SOCCER trial was performed based on the following assumptions. MSI is assumed to be 60 ± 20% [38–41] in the standard treatment group, also known as the O_2_ group. Inclusion of a total sample of 100 patients in the study, allows for the detection of an MSI difference of 15% between the O_2_ group and the air group with a power of 96% at a 5% risk of a Type I error. Since the effect of the culprit vessel on the effectiveness of the O_2_ therapy was considered a secondary outcome, no formal power calculation was conducted for this outcome.

## Results

**Fig. 1:**
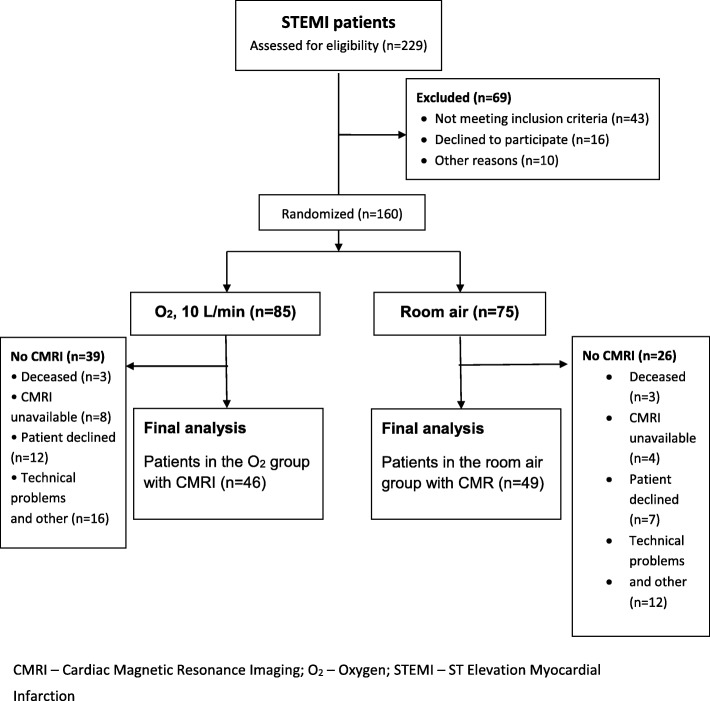
Patient flow diagram

Figure 1 displays the study profile. Of the 229 patients screened, 160 were randomized to either the supplemental O_2_ group (*n* = 85) or to the air group (*n* = 75). Of the included patients in the two arms, 54% (*n* = 46) and 65% (*n* = 49) respectively underwent a CMRI.

The majority of the patients included were male, and most of the included patients either smoked or were previous smokers (Table 1). The mean duration of the study intervention for both groups were 85 min, as symptom debut to PCI were on average 161 min (the air group) and 175 min (the O_2_ group). There were no significant differences in oxygen saturation at the time of randomization, while the oxygen saturation was higher in the O_2_ group during the intervention (99.2% vs 97.0%, *p* < 0.001). There were no other significant differences in baseline characteristics between the two groups, but a somewhat higher proportion of patients in the air group had diabetes and hypertension, while symptom debut to ambulance arrival/PCI times were somewhat higher in the O_2_ group.

**Table 1:** Patient and study characteristics for Cohort 1

Characteristics	O_2_ group (*n* = 46)	Air group (*n* = 49)	*P*-value
Demographics			
Male sex [n (%)]	29 (63.0)	34 (69.4)	NS
Age (year) [mean (SD)]	63.7 (13.1)	65.5 (11.5)	NS
BMI [mean (SD)]	26.1 (3.4)	27.0 (4.2)	NS
Smoker^a^ [n (%)]	31 (67.4)	33 (67.3)	NS
Medical history [n (%)]			
Diabetes	3 (6.5)	9 (18.4)	NS
Hypertension	11 (24.0)	21 (43.0)	NS
Previous stroke/TIA	0 (0)	3 (6.1)	NS
Previous medication [n (%)]			
ACEi	8 (17.4)	5 (10.2)	NS
Anticoagulant	1 (2.2)	0 (0)	NS
ARBs	2 (4.3)	2 (4.1)	NS
Aspirin	6 (13.0)	3 (6.1)	NS
β-blocker	0 (0)	7 (14.3)	NS
CCB	1 (2.2)	7 (14.3)	NS
Diuretics	1 (2.2)	7 (14.3)	NS
Statins	2 (4.3)	5 (10.2)	NS
Process times (min) [mean (SD)]			
Symptom to ambulance arrival	110.9 (112.6)	98.2 (87.8)	NS
Symptom to PCI	175.9 (121.6)	161.3 (93.1)	NS
Patient’s home to PCI	39.4 (11.2)	37.4 (10.4)	NS
Duration of study intervention (O_2_ or room air)			
Time (min) [mean (SD)]	85.6 (27.7)	85.4 (25.8)	NS
Intervention not for entire duration [n (%)]	3 (6.5)^b^	5 (10.2)^c^	NS
Vital parameters at inclusion			
Heart rate (BPM) [mean (SD)]	84.2 (17.3)	82.7 (18.2)	NS
Blood pressure (mmHg) [mean systolic pressure (SD)]	154.0 (29.6)	153.7 (29.1)	NS
Blood pressure (mmHg) [mean diastolic pressure (SD)]	94.7 (17.0)	91.4 (20.0)	NS
Blood oxygen saturation [mean (SD)]	98.0 (1.7)	97.7 (1.6)	NS
Vital parameters at arrival to the PCI laboratory			
Heart rate (BPM) [mean (SD)]	74 (13.1)	75 (18.9)	NS
Blood pressure (mmHg) [mean systolic pressure (SD)]	142.3 (22.2)	140.4 (23.8)	NS
Blood pressure (mmHg) [mean diastolic pressure (SD)]	85.3 (15.3)	84.8 (14.6)	NS
Blood oxygen saturation [mean (SD)]	99.2 (1.1)	97.0 (1.9)	< 0.001
Killip class [n (%)]			
Class I	45 (97.8)	48 (98.0)	NS
Class II	1 (2.2)	1 (2.0)	NS

*ACEi* Angiotensin Converting Enzyme Inhibitor, *ARBs* Angiotensin II Receptor Blockers, *BMI* Body Mass Index, *BPM* Beats Per Minute, *CCB* Calcium Channel Blocker, *NS* Not Significant, *PCI* Percutaneous Coronary Intervention, *TIA* Transitory Ischemic Attack

^a^Both current and past smoker

^b^The group allocation was unclear to the PCI personnel in two cases and in one case, the O_2_ therapy was terminated because the patient had chronic obstructive pulmonary disease

^c^All patients received supplemental O_2_ due to fall in O_2_ saturation < 94%

The majority in both groups had a single vessel disease, and close to a quarter of the included patients in both groups had a thrombectomy (Table 2). There were no significant differences between the two groups with regard to the culprit vessel, with LAD being the most common culprit vessel in both the O_2_ group (*n* = 23, 50%) as well as the air group (*n* = 23, 46.9%), followed by the RCA. Only four patients in the O_2_ group and three patients in the air group had the LCx as the culprit vessel.

**Table 2:** PCI laboratory procedural characteristics for Cohort 1

Characteristics	O_2_ group (*n* = 46)	Air group (*n* = 49)	*P*-value
Medications given [n (%)]			
IV β-blocker	3 (6.5)	5 (10.2)	NS
IV diuretics	3 (6.5)	6 (12.2)	NS
IV inotropes	0 (0)	1 (2.0)	NS
IV nitrate	3 (6.5)	2 (4.1)	NS
Culprit lesion [n (%)]			
Right Coronary Artery	18 (39.1)	20 (40.8)	NS
Left Anterior Descending Artery	23 (50.0)	23 (46.9)	NS
Left Circumflex Artery	4 (8.7)	3 (6.1)	NS
Other	1 (2.2)^a^	3 (6.1)^b^	NS
Coronary disease [n (%)]			
Single vessel	25 (54.3)	29 (59.2)	NS
Multivessel	20 (43.4)	17 (34.7)	NS
Left main coronary artery	1 (2.2)	3 (6.1)	NS
Procedures [n (%)]			
Thrombectomy	11 (24.0)	13 (26.5)	NS
CABG	2 (4.4)	2 (4.0)	NS

*CABG* Coronary Artery Bypass Grafting, *IV* Intravenous, *SC* Subcutaneous

^a^Culprit was the intermediate artery

^b^Culprit in one case was the intermediate artery and in two cases the D1 branch of the LAD

Table 3 shows the results of the CMRI for the both randomization groups. There was no significant difference in MSI among patients with a LAD culprit depending on allocation to O_2_ or air (47.8% vs 43.0%; *p* = 0.517). There were also no significant differences with regards to MaR, IS or hs-cTnT levels. Among the patients with a non-LAD culprit, there were no significant differences between the O_2_ or air group for neither the primary nor the secondary outcomes.

**Table 3:** CMRI results and the mean peak Troponin T in relation to the culprit lesion

Measures [mean (SD)]	O_2_ group (*n* = 46)		Air group (*n* = 49)		*P* -value for the difference	
	LAD (*n* = 23)	Non-LAD (*n* = 23)	LAD (*n* = 23)	Non-LAD (*n* = 26)	LAD	Non-LAD
MaR % of LV	36.3 (10.8)	27.5 (6.7)	36.9 (12.1)	23.9 (7.4)	0.717	0.081
MSI %	47.8 (24.0)	60.0 (25.2)	43.0 (24.1)	54.9 (22.8)	0.517	0.568
IS % of LV	19.4 (10.7)	11.9 (8.9)	19.4 (10.7)	10.9 (7.0)	0.448	0.787
IS ml	26.2 (17.3)	15.1 (11.5)	26.2 (17.3)	13.3 (9.4)	0.733	0.696
hs-cTnT [median (IQR)]	3319.0 (5619.0)	2760.0 (3875.0)	3862.0 (7103.0)	922.5 (2793.3)	0.717	0.062

*CMRI* Cardiac Magnetic Resonance Imaging, *hs-cTnT* High-Sensitive Cardiac Troponin T, *IS* Infarct Size, *IQR* Interquartile range, *LV* Left Ventricle, *MaR* Myocardium at Risk, *ml* Milliliters, *MSI* Myocardial Salvage Index, *O*_*2*_ Oxygen, *SD* Standard Deviation

**Fig. 2:**
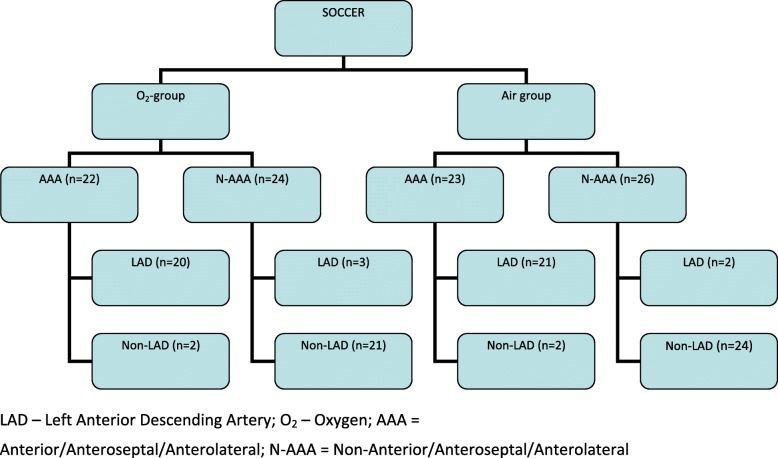
ECG findings with respect to the culprit lesion

Figure 2 shows the corresponding angiographic culprit vessel depending on the ECG pattern. As shown in Table 4, there were no significant differences with regards to the primary outcome between patients randomized to O_2_ or air based on the ECG STEMI pattern. There were also no significant differences for the secondary outcomes, except for MaR which was significantly larger among N-AAA patients randomized to the O_2_ group vs air (29.2% vs 23.7%; *p* = 0.021).

**Table 4:** CMRI results and the median peak Troponin T in relation to the ECG findings

Measures [mean (SD)]	O_2_ group (*n* = 46)		Air group (*n* = 49)		*P* -value for the difference	
	AAA (*n* = 22)	N-AAA (*n* = 24)	AAA (*n* = 23)	Non-AAA (*n* = 26)	AAA	N-AAA
MaR % of LV	34.8 (10.8)	29.2 (8.4)	37.1 (11.9)	23.7 (7.5)	0.401	*0.021*
MSI %	48.8 (25.3)	58.6 (24.5)	47.9 (26.9)	50.6 (21.3)	0.964	0.286
IS % of LV	18.6 (11.2)	13.0 (9.1)	20.7 (13.0)	11.8 (6.6)	0.482	0.641
IS ml	25.0 (17.5)	16.6 (12.6)	26.8 (19.2)	14.2 (9.1)	0.803	0.560
hs-cTnT [median (IQR)]	3487.5 (6035.3)	3039.0 (3705.8)	3862.0 (7458.0)	1079.0 (2726.5)	0.910	0.074

*AAA* Anterior/Anteroseptal/Anterolateral, *CMRI* Cardiac Magnetic Resonance Imaging, *hs-cTnT* High-Sensitive Cardiac Troponin T, *IS* Infarct Size, *IQR* Interquartile range, *LV* Left Ventricle, *MaR* Myocardium at Risk, *ml* Milliliters, *MSI* Myocardial Salvage Index, *O*_*2*_ Oxygen, *N-AAA* Non-Anterior/Anteroseptal/Anterolateral, *SD* Standard Deviation

We also evaluated the effect of supplemental O_2_ therapy by subgroup with respect to both the culprit vessel (Table 5) and ECG finding (Table 6), using multiple regression with an interaction term. No significant effect of O_2_ therapy was found in any group for any outcome.

**Table 5:** CMRI Linear Regression and the culprit vessel

Variables	MSI %		MaR % of LV		IS % of LV		IS ml		hs-cTnT	
	Coefficient (CI)	*p*-value	Coefficient (CI)	*p*-value	Coefficient (CI)	*p*-value	Coefficient (CI)	*p*-value	Coefficient (CI)	*p*-value
Effect of O_2_ in LAD-group	4.82 (−9.23;18.87)	0.497	−0.66 (−6.22;4.89)	0.813	−2.26 (−7.96;3.45)	0.434	−1.65 (−10.13;6.83)	0.700	− 584.4 (− 2502.9;1334.1)	0.547
Effect of O_2_ in non-LAD-group	5.09 (−8.55;18.73)	0.460	3.58 (−1.81;8.98)	0.190	0.97 (−4.57;6.50)	0.729	1.79 (−6.45;10.01)	0.668	1039.0 (− 823.3;2901.3)	0.271

*hs-cTnT* High sensitive cardiac troponin T, *IS* Infarct Size, *LAD* Left Anterior Descending artety, *LV* Left Ventricle, *MaR* Myocardium at Risk, *MSI* Myocardial Salvage Index, *O*_*2*_ Oxygen

**Table 6:** CMRI Linear regression and ECG findings

Variables	MSI %		MaR % of LV		IS % of LV		IS ml		hs-cTnT	
	Coefficient (CI)	*p*-value	Coefficient (CI)	*p*-value	Coefficient (CI)	*p*-value	Coefficient (CI)	*p*-value	Coefficient (CI)	*p*-value
Effect of O_2_ in AAA-group	0.90 (−13.60;15.41)	0.902	−2.298 (− 8.06;3.47)	0.430	−2.17 (− 8.17;3.84)	0.475	− 1.77 (− 10.61;7.08)	0.693	− 474.4 (− 2427.1;1478.4)	0.631
Effect of O_2_ in N-AAA-group	8.03 (−5.74;21.79)	0.250	5.44 (−0.03;10.92)	0.051	1.23 (− 4.47;6.93)	0.669	2.38 (− 6.01;10.78)	0.077	962.8 (− 890.8;2816.4)	0.305

*AAA* Anterior/Anteroseptal/Anterolateral, *hs-cTnT* High sensitive cardiac troponin T, *IS* Infarct Size, *LAD* Left Anterior Descending artety, *LV* Left Ventricle, *MaR* Myocardium at Risk, *MSI* Myocardial Salvage Index, *O*_*2*_ Oxygen

## Discussion

**Fig. 3:**
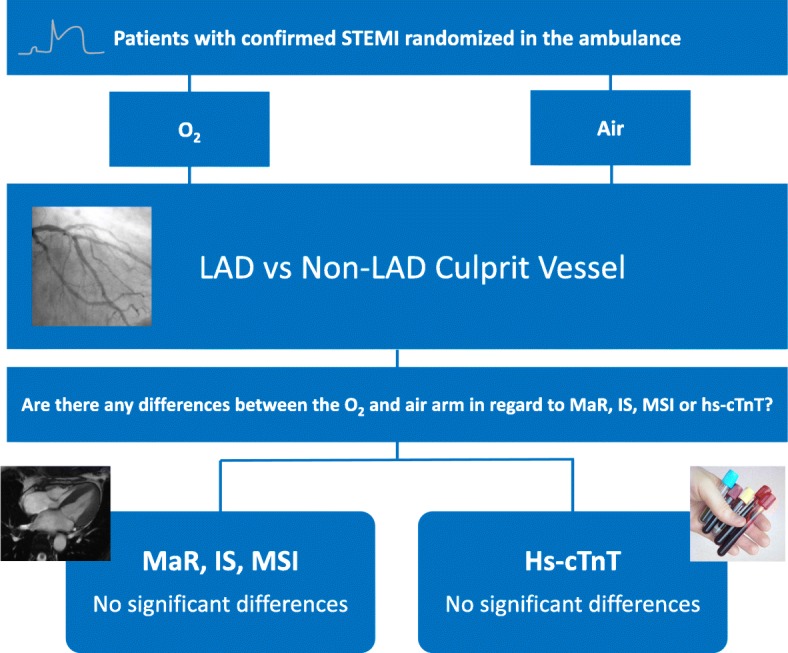
Central Illustration

In this sub-study of the SOCCER trial, we evaluated the effects of supplemental O_2_ therapy in STEMI patients with regard to MSI, MaR, and IS based on the culprit vessel (Fig. 3). Our main finding was that there is no significant difference between the patients randomized to the O_2_ group or the air group based on the location of the culprit vessel as identified by the interventional cardiologist performing the coronary angiography. The same results could also be shown based on the ECG STEMI pattern. Although MaR was significantly larger in the O_2_ group among N-AAA patients, this is somewhat hard to explain on a theoretical basis and likely due to chance alone.

In the AVOID trial [14] in which 441 STEMI patients were randomized to O_2_ therapy or air, there was a significant increase in Creatine Kinase (CK) levels in the O_2_ group, with a similar trend shown among STEMI patients with an LAD and non-LAD culprit. In our study we however did analysis based on culprit vessel using MaR, IS, and MSI as measured by CMR, which are superior to CK levels for assessing infarct size and myocardial injury as well as prognosis [38, 42–44], and could not see any significant differences between the O_2_ and air groups.

The DETO2X-AMI trial conducted in Sweden had all-cause mortality at 1 year as the primary outcome and included more than 6000 patients with suspected MI who were randomized to either O_2_ therapy or air; the trial showed no difference between the groups [4]. A sub-study of the trial focusing on STEMI patients (*n* = 2807) randomized to O_2_ therapy or air, also showed no differences between the groups in regard to one-year all-cause mortality or morbidity [8]. They did not however evaluate whether there were any differences based on culprit vessel.

In our previous publications [9, 10, 28], we have evaluated the role of supplemental O_2_ in first time STEMI patients with respect to MSI, MaR and IS as measured by Cardiac Magnetic Resonance Imaging (CMRI), and Wall Motion Score Index and Left Ventricular Ejection Fraction as measured by Echocardiography [10, 28]. The results, in line with other trials, showed no negative nor positive effects of supplemental O_2_ therapy in regard to the studied outcomes. We have also previously shown that there does not seem to be a significant analgesic effect of O_2_ in patients with STEMI [9].

These findings were confirmed in a recent meta-analysis including eight RCTs studying the effects of supplemental O_2_ therapy in patients with suspected and confirmed MI (*n* = 7998), showing no clinical benefits [45]. In another meta-analysis including 25 RCTs and a total study population of 16,037 acutely ill patients, it was shown that supplemental O_2_ therapy in normoxemic (blood O_2_ saturation ≥ 94%) patients increased mortality [46]. It is thus clear from the current evidence that supplemental O_2_ therapy should only be considered in patients with hypoxemia.

Our results are thereby in line with previous studies showing no positive nor negative effects of supplemental O_2_ therapy in patients with confirmed or suspected MI, [3, 4, 6, 8–11, 13, 45] and this also seems to be the case irrespective of culprit vessel. It thereby seems safe to withhold oxygen in normoxic STEMI patients, irrespective of infarct location.

## Limitations

Our results are only applicable to STEMI patients and not acute coronary syndrome patients as a whole as patients with non-STEMI and unstable angina were not included. Furthermore, since our results are from two centers only, they may not be generalizable to other settings. The included patients were also normoxic and hemodynamically stable, why our results may not be applicable to unstable STEMI patients. After allocating the included patients into different culprit vessel subgroups, the number of patients studied in each group were few why there may be some risk of type 2 error.

Since our primary outcome (MSI) and most of the secondary outcomes (MaR and IS) were solely based on patients undergoing CMRI, a drop-out of patients was inevitable. Although the dropout of patients may be a source of bias, the reasons for why some patients did not undergo CMRI were almost equal between the two groups (Fig. 1).

## Conclusion

There were no significant differences in MSI, MaR, IS, or hs-cTnT levels between first time STEMI patients randomized to supplemental O_2_ therapy or air, in relation to whether the culprit vessel was the LAD or not. Our results thereby indicate that the location of the culprit vessel probably has no effect on the role of supplemental O_2_ therapy in STEMI patients.

## Data Availability

A de-identified dataset will be made available upon written reasonable request to the corresponding author.

## Abbreviations

AAA: Anterior/Anteroseptal/Anterolateral
CCU: Cardiac Care Unit
CMR: Cardiac Magnetic Resonance
CMRI: Cardiac Magnetic Resonance Imaging
ECG: Electrocardiogram
hs-cTnT: High Sensitive Troponin-T
IS: Infarct Size
LAD: Left Anterior Descending artery
MaR: Myocardium at Risk
MSI: Myocardial Salvage Index
N-AAA: Non-Anterior/Anteroseptal/Anterolateral
O_2_: Oxygen
PPCI: Primary Percutaneous Coronary Intervention
RCT: Randomized Controlled Trials
STEMI: ST Elevation Myocardial Infarction
